# Comparison of sEMG-Based Feature Extraction and Motion Classification Methods for Upper-Limb Movement

**DOI:** 10.3390/s150409022

**Published:** 2015-04-16

**Authors:** Shuxiang Guo, Muye Pang, Baofeng Gao, Hideyuki Hirata, Hidenori Ishihara

**Affiliations:** 1 The Institute of Advanced Biomedical Engineering System, School of Life Science and Technology, Beijing Institute of Technology, Haidian District, Beijing 100081, China; E-Mails: guoshuxiang@hotmail.com (S.G.); gaobaofeng@bit.edu.cn (B.G.); 2 Key Laboratory of Convergence Medical Engineering System and Healthcare Technology, The Ministry of Industry and Information Technology, School of Life Science and Technology, Beijing Institute of Technology, Haidian District, Beijing 100081, China; 3 Faculty of Engineering, Kagawa University, Hayashi-cho, Takamatsu 761-0369, Japan; E-Mails: hhirata@eng.kagawa-u.ac.jp (H.H.); ishihara@eng.kagawa-u.ac.jp (H.I.)

**Keywords:** surface electromyography, motion recognition, muscular model, weight peaks, neural networks, support vector machine

## Abstract

The surface electromyography (sEMG) technique is proposed for muscle activation detection and intuitive control of prostheses or robot arms. Motion recognition is widely used to map sEMG signals to the target motions. One of the main factors preventing the implementation of this kind of method for real-time applications is the unsatisfactory motion recognition rate and time consumption. The purpose of this paper is to compare eight combinations of four feature extraction methods (Root Mean Square (RMS), Detrended Fluctuation Analysis (DFA), Weight Peaks (WP), and Muscular Model (MM)) and two classifiers (Neural Networks (NN) and Support Vector Machine (SVM)), for the task of mapping sEMG signals to eight upper-limb motions, to find out the relation between these methods and propose a proper combination to solve this issue. Seven subjects participated in the experiment and six muscles of the upper-limb were selected to record sEMG signals. The experimental results showed that NN classifier obtained the highest recognition accuracy rate (88.7%) during the training process while SVM performed better in real-time experiments (85.9%). For time consumption, SVM took less time than NN during the training process but needed more time for real-time computation. Among the four feature extraction methods, WP had the highest recognition rate for the training process (97.7%) while MM performed the best during real-time tests (94.3%). The combination of MM and NN is recommended for strict real-time applications while a combination of MM and SVM will be more suitable when time consumption is not a key requirement.

## 1. Introduction

With the development of electromyography (EMG) techniques, the surface EMG (sEMG) signal, which reflects the activation level of skeletal muscles, has become an advanced tool for intuitive control of prostheses or robot arms. Fukuda *et al*. [1] used sEMG signals to control a manipulator. They adopted a statistical neural network, named the log-linearized Gaussian mixture network, to achieve robust discrimination between differences among individuals, electrode locations, and time variations caused by fatigue or sweat. They reported that the method can provide smooth control for the manipulator and it might allow a physically handicapped person to sense a feeling of prosthetic control similar to that of the original limb. Liarokapis *et al*. [2] used sEMG signals from sixteen muscles of the upper limb to study the muscular co-activation patterns during a variety of reach-to-grasp motions. Chen *et al*. [3] developed a multi-kernel learning support vector machine method to classify multiple finger movements. In our previous study, an AR model-based multi-motion recognition method was proposed to classify different upper-limb motions to control an upper-limb exoskeleton rehabilitation device [4,5].

One of the main factors preventing the implementation on EMG signals outside a laboratory environment is the unsatisfactory motion recognition accuracy rate based on EMG signals. Many efforts have been made in order to improve the recognition accuracy rate. Ju *et al*. [6] designed a fuzzy Gaussian mixture model with a non-linear feature extraction method to classify different hand grasps and in-hand manipulations. They reported that by using their proposed non-linear method a recognition rate of 96.7% could be achieved. Artemiadis *et al*. [7] developed a switching regime model to decode the EMG activity from 11 muscles into a continuous representation of arm motion in three-dimensional space. They reported that this switching regime model could overcome some of the main difficulties of EMG-based control systems, such as the nonlinearity between the EMG recordings and the arm motion, as well as the non-stationarity of EMG signals with respect to time. Balbinot and Favieiro [8] designed a neuro-fuzzy system to classify human upper-limb movements. Alkan and Gunay [9] used SVM to classify four upper limb movements. Moreover, Young *et al*. [10] provided a Bayesian theory based conditional parallel linear discriminant analysis (LDA) classifiers for simultaneous movement recognition. Different from regarding the combined movements (with multi-DoF) as independent motions, the proposed classifier could recognize the simultaneous movements with a single-DoF training set.

One of the problems associated with misclassification is the cross-talk, which results from the complex musculoskeletal structure. Shibanoki *et al*. [11] designed a quasi-optimal channel selection method to select the effective channels for recognition of 16 hand gestures. Naik and Kumar [12,13] applied blind source separation algorithms to separate the more useful information from cross-talk signals to improve the finger-movement recognition accuracy rate. In later research, Naik and Nguyen [14] applied non-negative matrix factorization to identify finger movements and also used independent component analysis to evaluate myoelectric sensor placement [15]. Zhang *et al*. [16] developed a hand gesture recognition system using not only EMG signals, but also accelerometer information, to identify 72 Chinese sign language words. Tang *et al*. [17] applied a multi-channel energy ratio feature extraction method to overcome the influence of various forces for a given gesture. They used the proposed feature extraction method and a Cascaded-Structured classifier to recognize eleven hand gestures. Rafiee *et al*. [18] proposed a Mother Wavelet Matrix (MWM), which has the potential to differentiate the feature vectors to extract features from sixteen locations of EMG signals to classify ten hand motions. For these recognition solutions, many elegant feature extraction or motion classification methods have been proposed based on mathematics or signal processing points of views. As human motion is the consequence of the complex human musculoskeletal system, depending on the generalization of machine learning algorithm seems insufficient.

Besides motion recognition, EMG signals are also used for musculotendon force prediction. Two physiological models are widely used for musculotendon force predictions: Huxley- [19,20] and Hill-type models [21]. Compared with the complexity of Huxley-type models, Hill-type models are more computationally viable. Cavallaro *et al*. [22] developed a Hill-typemodel-based myoprocessor to predict joint torque. Seven muscles around the upper limb were recorded and a genetic algorithm was implemented to tune the parameters of the model. We also proposed a continuous elbow angle prediction method based on the Hill-type muscular model and a simplified musculoskeletal model [23]. This method can predict elbow joint angle continuously using only EMG signals recorded from the *biceps brachii* muscle. The motion was constrained in the sagittal plane. Nevertheless, it is relevant difficult to predict motions based on musculotendon forces because of the redundancy of the human musculoskeletal system. In order to simplify the problem, a constrained movement was required in most of the cases [24]. However, the muscular model provides a natural way with sufficient biological meaning for feature extraction for the EMG based motion recognition.

For intuitive control of robot arms, movements of upper limb in daily life are often used. The method applied based on sEMG signals should have the ability to recognize these motions. Furthermore, a real-time response for controlling is often requested. The purpose of this paper is to compare the performance of different recognition methods for eight upper limb motions (elbow flexion and extension, forearm pronation and supination, wrist flexion and extension, wrist abduction and adduction), with various combinations of four feature extraction (RMS, DFA, WP, MM) and two classification methods (NN and SVM), to determine their correlation and a proper way for motion recognition with sEMG signals. The time consumption for training is also discussed, as it reflects the performance of methods.

## 2. Methods

As introduced previously, four feature extraction methods and two classifiers were chosen for study in this paper. The description for these methods and classifiers are introduced in this Section. However, these methods were applied on pre-processed sEMG signals, which will be explained in the next Section.

### 2.1. The WPT-Based RMS Feature Extraction Method

The purpose of applying Wavelet Packet Transforms (WPT) in this study is to reduce the influence from extrinsic noises. Although a commercial filter box was used, noise during sampling is inevitable. Such noise can be introduced by relative movement between electrodes and surface skin, or sweat from the skin.

Assuming that *U_0_^0^* is the scaling space of an EMG signal *E*(*t*), WPT can decompose *U_0_^0^* into small subspaces in dichotomous way with the algorithm defined in Equation (1):

(1) $${U^{n}}_{j1}={U^{2n}}_{j}⊕{U^{2n+1}}_{j},j\in Z;n\in Z_{+}$$

where *j* is the resolution level and ⊕ is the orthogonal decomposition. *U^n^_j1_*, *U^2n^_j_* and *U^2n+1^_j_* are three close spaces corresponding to *u_n_*(*t*), *u_2n_*(*t*) and *u_2n+1_*(*t*). The relationship between *u_n_*(*t*), *u_2n_*(*t*) and *u_2n+1_*(*t*) is defined as following:

(2) $$u_{2n}(t)=\sqrt{2}\sum_{k\in Z}h(k)u_{n}(2t-k)$$

(3) $$u_{2n+1}(t)=\sqrt{2}\sum_{k\in Z}g(k)u_{n}(2t-k)$$

where the function *u_0_*(*t*) can be identified with the scaling function *φ* and *u*_1_(*t*) can be identified with a mother wavelet *Ψ*. *h*(*k*) and *g*(*k*) are the coefficients of the low-pass and the high-pass filters respectively. The sub-signal at *U*^n^*_j1_* on the jth level can be reconstructed by Equation (4):

(4) $${s^{n}}_{j}(t)=\sum_{k}{D^{j,n}}_{k}\psi_{j,k}(t),k\in Z$$

where *Ψ_j,k_*(*t*) is the wavelet function, *D^j,n^_k_* is the wavelet packet coefficients at *U^n^_j1_*, which can be calculated by Equation (5).

(5) $${D^{j,n}}_{k}=\int s(t)\psi_{j,k}(t)dt$$

In this paper, we chose Daubechies 2 and decomposition raw sEMG signal to the fourth level. The reconstructed wavelet signals calculated by Equation (5) were used to extract features for motion recognition. The Root Mean Square (RMS) method was then applied to extract features from the processed sEMG signals with WPT. RMS values were calculated using an overlapping window of 100 samples length with a 25 samples increment between windows.

### 2.2. WPT Based Weight Peaks Feature Extraction Method

Although the RMS method can represent the energy of the original signals, in our particular case the trends that reflect changes of muscle contraction are more suitable to represent the features for motion recognition. The purpose of the WP method is to capture the trend of the original sEMG signals [25]. The reconstructed sEMG signals processed by WPT have different frequencies in different nodes. Therefore, the amount of peaks obtained in different nodes is different. Zero crossing which is defined as Equation (6) is used to find where the peak exists:

(6) $$ZC=\sum_{n=1}^{N-1}\left(\mathrm{sgn}(s_{n}\times s_{n+1})\cap \left|s_{n}-s_{n+1}\right|\geq threshold\right)$$

All the reconstructed sEMG signals of zero crossing are saved to obtain peaks and valleys among them.

The procedure of the WP method is described as follows:

*If max*(*sZC*(*i*)*: sZC*(*i +* 1)) *+ min*(*sZC*(*i*)*: sZC*(*i +* 1)) *≥* 0

*P*(*i*) *= max*(*sZC*(*i*)*: sZC*(*i +* 1))

*Else if max*(*sZC*(*i*)*: sZC*(*i +* 1)) *+ min*(*sZC*(*i*)*: sZC*(*i +* 1)) *<* 0

*P*(*i*) *=* (*−*1) *× min*(*sZC*(*i*)*: sZC*(*i +* 1))

where *sZC*(*i*) is the reconstructed sEMG signal of zero crossing. *P*(*i*) is the peak or valley between the data of zero crossing and valleys are transformed into positive numbers.

During experiments, we found that the higher peaks reflect the trend of motion more than the lower peaks. Therefore the next step of weighted peaks is to increase the higher peak component and decrease the lower peak component to obtain a feature near to the trend of a subject’s motion. The algorithm is defined in Equation (7), where *n* is set experientially:

(7) $$P(i+1)=\frac{n-1}{n}P(i)+\frac{1}{n}P(i+1)$$

The overlapping window for WP (*N* in Equation (6)) dependeds on the threshold set to detect the zero crossing. According to the experimental results, the calculated features took about 90% of the total samples.

### 2.3. Detrended Fluctuation Analysis

After the introduction of DFA by Peng *et al*. [26], this method attracted a lot of attention for analyzing time series. It is believed that DFA is suitable for application to signals whose underlying statistics or dynamics are non-stationary; EMG signals are typical non-stationary.

The first step in DFA is to convert a bounded time series *x_t_* into an unbounded process *X_t_* using the following Equation (8):

(8) $$X_{t}=\sum_{i=1}^{t}\left(x_{i}-\bar{x_{i}}\right)$$

where $\bar{x_{i}}$ is the average value of *x_i_*.

*X_t_* is then divided into time windows *Y_j_* of *L* samples of length and local least squares straight-line fitting is calculated by minimizing the squared error with respect to slope and intercept parameters *a* and *b*:

(9) $$E^{2}=\sum_{j=1}^{L}{\left(Y_{i}-ja-b\right)}^{2}$$

Then fluctuation or RMS deviation from the trend is calculated over every window at every time scale:

(10) $$F(L)={\left(\frac{1}{L}\sum_{j=1}^{L}{\left(Y_{j}-ja-b\right)}^{2}\right)}^{1/2}$$

This detrending followed by fluctuation measurement process is repeated over the complete signals set. The disjoint window for DFA was 100 samples.

### 2.4. Muscular Model Based Feature Extraction Method

The Hill-type based muscular model has already been applied successfully in our previous study to predict elbow joint angles [23]. The advantage of this method is that it can provide quantitative relationships between sEMG signals and elbow joint angles based on a biological point of view, not just considering the issue as a single processing problem. The Equations used to describe Hill-type model are:

(11) $$F_{PE,SE}=F_{\max}(\exp(S\Delta L/\Delta L_{\max})-1)/(\exp(S)-1)$$

(12) $$F_{CE}=F_{\max}u\cdot f_{l}\cdot f$$

(13) $$f_{v}=0.1433{\left(0.1074+\exp\left(-1.3\sinh\left(2.8V_{CE}/V_{CE_{0}}+1.64\right)\right)\right)}^{-1}$$

(14) $$f_{v}=0.1433{\left(0.1074+\exp\left(-1.3\sinh\left(2.8V_{CE}/V_{CE_{0}}+1.64\right)\right)\right)}^{-1}$$

(15) $$V_{CE_{0}}=0.5\left(u+1\right)V_{CE_{\max}}$$

(16) $$F_{T}=F_{CE}+F_{PE,SE}$$

where SE denotes the passive serial element, CE denotes the active contractile element and PE denotes the passive element arranged in parallel to the previous two. Δ*L* is the change in length of the element with respect to the slack length, *f_l_* is the factor of force introduced by the changes of muscle length and *f_v_* is another factor of force introduced by changes of muscle change velocity. *S* is a shape parameter, *F*_max_ is the maximum force exerted by the element for the maximum change in length Δ*L*_max_, and *F_PE,SE_* is the passive force generated by the PE or SE depending on the set of parameters used. *F_T_* is the total force exerted by the muscle. *u* is the activation level of a muscle. For simplicity, Δ*L* was considered as a constant and *V_CE_* is zero. The effect of SE and PE was also ignored. Therefore, the musculotendon force (*F_T_*) has a relative simple relationship with the muscle activation level (*u*) under these certain conditions.

Furthermore, raw sEMG signals should be filtered by a high-pass filter to remove any DC offsets or low-frequency noise and then rectified. Sometimes, these rectified signals are directly transformed into muscle activation levels by dividing them by the peak rectified EMG value obtained during the MVC test. Some researchers [27] suggest that a more detailed model of muscle activation dynamics is warranted in order to characterize the time-varying features of the sEMG signal. In this paper, a discretized recursive filter was used.

A discretized recursive filter with a continuous form of a second-order differential equation was implemented:

(17) $$u(t)=Md^{2}e(t)/d^{2}t+Bde(t)/dt+Ke(t)$$

where *M*, *B*, and *K* are the constants that define the dynamics of Equation (17) and *e*(*t*) is the processed sEMG signal. This equation can be expressed in discrete form using backward differences:

(18) $$u(t)=\alpha e(t-d)-\beta_{1}u(t-1)-\beta_{2}u(t-2)$$

where *d* is the electromechanical delay and *α*, *β*_1_, and *β*_2_ are the coefficients that define the second-order dynamics. Selection of the values for *α*, *β*_1_, and *β*_2_ should follow the following restrictions:

(19) $$\beta_{1}=\gamma_{1}+\gamma_{2}$$

(20) $$\beta_{2}=\gamma_{1}\times \gamma_{2}$$

(21) $$\left|\gamma_{1}\right|<1$$

(22) $$\left|\gamma_{2}\right|<1$$

(23) $$\alpha -\beta_{1}-\beta_{2}=1$$

In order to guarantee the stability of the equation and that neural activation does not exceed 1, the calculation results should be filtered by a low-pass filter (with a cut-off frequency of 3–10 Hz) because the muscle naturally acts as a filter. The resulting force changing frequency is much lower than the amplitude changing frequency of sEMG signals. The MM feature extraction method was applied on every recorded sample in real-time. To summarize, the numbers of features generated by the four feature extraction methods in 1 s, given the condition of 1000 Hz sampling frequency, are listed in Table 1.

**Table 1:** Feature numbers for the four extraction methods.

	Feature Extraction Method			
****Feature Numbers****	RMS	WP	DFA	MM
	40	≈900	10	1000

The four feature extraction methods were applied on every sEMG recording channel and the calculated features from all the channels were used as inputs simultaneously for the classifiers.

### 2.5. Classification Method

The Neural Network (NN) classifier and Support Vector Machine (SVM) classifier were used to recognize the motions. The activation function of the NN is described as:

(24) $$f(s)=1/(1+e^{-\mu s})$$

where *μ* is a constant coefficient and *s* is the summation of the input defined as:

(25) $$s=\sum w_{i}x_{i}$$

where *w_i_* is a weighted parameter for each input to the neural node. There is one hidden layer with eight neurons in the neural network. The hyperbolic tangent sigmoid transfer function and scaled conjugate gradient backpropagation training algorithm are adopted for hidden layer combination and learning process, respectively. The dimension of the input vector equals the number of muscles used for sEMG signals recording. The output are vectors structured by one-of-k coding scheme where k is the number of patterns (in this paper it is 9, including the relax motion).

SVM is proposed as a classification technique based on maximizing the margin between a data set and using an optimal hyper plane to separate different data sets [28]. From a mathematial point of view, SVM requires solving the following optimization problem to find the minimum of Equation (26):

(26) $$\varphi (\omega ,\xi )=0.5\left\|\omega^{2}\right\|+c\sum_{i=1}^{l}\xi_{i}$$

subject to:

(27) $$y_{i}[(x_{i}\cdot \omega )+b]\geq 1-\xi_{i},i=1,2,...,l$$

where *x* is an n-dimensional vector (in this paper it is 6-dimensional as there are six independent inputs) and *b* is a scalar. *c* is the independent variable. *l* is the number of data points. *y* is the model to be learned. Equations (26) and (27) can be rewritten in the dual Lagrangian form:

(28) $$\tilde{L}(a)=\sum_{n=1}^{l}a_{n}-0.5\sum_{n=1}^{l}\sum_{m=1}^{l}a_{n}a_{m}t_{n}t_{m}k(x_{n},x_{m})$$

where *k*(*x_n_*,*x_m_*) denotes the kernel function which plays the soul role in SVM. Compared with neural network, the objective function of SVM is convex, giving rise in the relatively straightforward solution of the optimization problem.

To classify new data using the trained model, Equation (29) is used based on the conception of kernel function:

(29) $$\text{y}(x)=\sum_{n=1}^{N}a_{n}t_{n}k(x,x_{n})+b$$

where *N* denotes the number of support vectors. The value of target *y* is correspond to the target motions. In this paper it is integer ranged from 1 to 9.

One of the most popular approaches to training SVM is the Sequential Minimal Optimization (SMO) algorithm [29] which solves the quadratic programming problem of training a SVM. The details for SVM can be found in [30]. As there were four feature extraction methods and two classifiers, eight combinations (4 × 2) were tested in the following experiments, which were RMS + NN, RMS + SVM, WP + NN, WP + SVM, DFA + NN, DFA + SVM, MM + NN and MM + SVM. The classification was performed on data recorded continuously during the tasks.

## 3. Experimental Results

### 3.1. Experimental Setup

**Figure 1:**
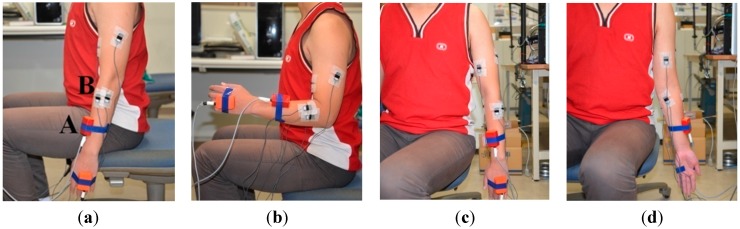
(**a**) shows the gesture of relaxing where A is the MTx sensor and B is the electrode; (**b**) shows the gesture of elbow extension; (**c**) shows the gesture of forearm pronation; (**d**) shows the gesture of forearm supination.

**Figure 2:**
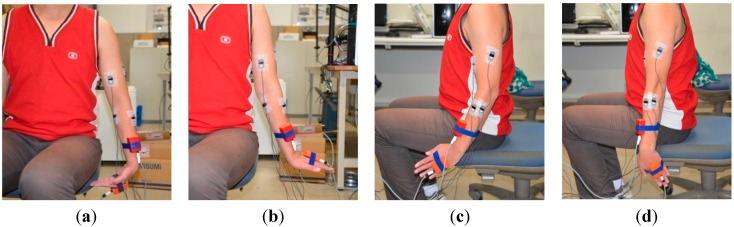
(**a**) shows the gesture of wrist flexion; (**b**) shows the gesture of wrist extension; (**c**) shows the gesture of wrist abduction; (**d**) shows the gesture of wrist adduction.

Seven healthy volunteers (aged from 22 to 28, median 25.70 ± 1.61 years, all male, two left handed and five right handed, height: 1.71 ± 0.17 m, weight: 65.66 ± 8.14) participated in the experiments. In the elbow flexion and extension experiments, subjects were asked to sit on a chair starting with their upper limbs relaxed vertically in the sagittal plane and then flex their forearms to 90°. After having maintained their forearms in a horizontal position for 2 s, the subjects were asked to extend their forearms to the initial vertical position (as shown in Figure 1a,b). In the forearm pronation and supination experiments, subjects were asked to relax their upper limbs vertically and only pronate and supinate forearms to the maximum degrees as they felt comfortable, keeping the upper arm still (as shown in Figure 1c,d). In the experiments on wrist flexion and extension, subjects relaxed their upper limbs vertically and flexed and extended their wrists to the maximum degree as they felt comfortable (as shown in Figure 2a,b). In the wrist adduction and abduction experiments, subjects again relaxed their upper limbs vertically as they did in the previous two experiments, and performed the abduction and abduction movement as shown in Figure 2c,d).

Each subject repeated the four experiments five times to acquire enough data to train the classifiers. After classifier training, another five trials were conducted for each of the four experiments to test the on-line performance of the recognition methods. All subjects practiced several times following a standard video before experiments.

*Flexor carpi radialis* (FCR), *flexor carpi ulnaris* (FCU), *extensor carpi radialis* (ECR), *extensor carpi ulnaris* (ECU), *biceps brachii* (BB) and *triceps brachii* (TB) were selected to record sEMG signals. All six s of these sEMG data channelwere used simultaneously for the recognition of the eight motions. Prior to data collection, the skin was shaved and wiped with alcohol. Dry rectangle electrodes (Ag/AgCl, size: 26 × 14 mm), with a skin contact surface of 20 mm^2^ and inter-electrode distance of 15 mm, were placed parallel to the muscle fibers, according to SENIAM references [31]. Electrode placements were confirmed by voluntary muscle contraction and followed the recommendation of [32]. For FCR, the electrode placement is one-third the distance from the medial epicondyle to the radial styloid. For FCU, it is one-third of the forearm from the medial prominence. For ECR, it is one-third the distance from the lateral epicondyle to the wrist. For ECU, it is one-third of the forearm along the ulna. For BB, it is at the middle of superficial muscle belly. For TB, it is at the long head position. The sampling rate for sEMG signals was 1000 Hz with differential amplification (gain: 1000) and common mode rejection (104 dB) by the commercial filter box (10–450 Hz band-pass, 60 Hz notch filtered, Personal EMG, Oisaka Development Ltd., Fukuyama, Japan). The filtered sEMG signals were then rectified and normalized by MVC values. Two MTx sensors (Xsens Technologies B.V., Enschede, The Netherlands) were attached to the subject’s hand and forearm to record the joint and wrist angles, which were used for labeling the training data. NN and SVM were trained by MATLAB Neural Networks Recognition Tools (MATLAB NRT, MathWorks Co., Natick, MA, USA) and LIBSVM [33], respectively. The on-line tests were conducted using self-designed software developed by Microsoft Visual Studio 2010 (Microsoft Co., Redmond, WA, USA) based on MATLAB NRT and LIBSVM.

### 3.2. Experimental Results

Different combinations of the four feature extraction methods and two classifiers during the training process are listed in Table 2. For the performance of recognition accuracy rate, the results show that the combination of WP and NN obtained the highest rate (with an average of 97.6%) and the combination of RMS and SVM obtained the lowest rate (with an average of 73.1%). Among the feature extraction methods, the performance of WP and MM is better than that of RMS and DFA while WP is a little better than MM (97.6% *vs.* 95.1%). Between the two classifiers, NN performs better than SVM during training process for all four features.

**Table 2:** Performance of off-line training. The value is the recognition accuracy rate defined by number of corrected recognized results dividing total results, with unit of %.

	Subject (NN/SVM)							
Features	A	B	C	D	E	F	G	Average
RMS	75.2/74.2	70.7/66.8	82.2/80.5	81.2/78.9	76.1/71.5	70.1/69.7	73.3/70.1	75.5/73.1
RMSF	87.0/82.0	84.1/83.5	90.6/89.5	89.6/86.8	86.5/85.4	82.1/80.2	85.1/81.2	86.4/84.0
WP	98.4/97.6	97.2/94.1	97.7/94.5	98.9/96.8	97.5/95.2	97.5/94.5	96.5/92.5	97.7/95.0
MM	98.8/98.0	95.7/90.9	94.1/91.8	91.4/88.3	93.1/90.14	95.3/93.4	97.3/95.4	95.1/92.6

Experimental results of on-line performance are listed in Table 3. It can still be observed that WP and MM obtain higher recognition accuracy rates than RMS and DFA, while MM is a little higher than WP (94.3% *vs*. 92.0%). However, the performance of NN is worse than that of SVM during on-line testing experiments (except for subject D). The recognition accuracy rates of both classifiers decrease compared with the performance during training period. The magnitude of this decrease for NN is larger than that of SVM.

Among the eight motions, forearm pronation and supination are different from the others as the muscles used to recognize these two motions are not directly involved. The recognition results are listed in Table 4. Although some of these recognition accuracy rates were relative low, all the recognition accuracy rates were above 90% (with the combination of WP + SVM).

**Table 3:** Performance of on-line testing. The value is the recognition accuracy rate defined by number of corrected recognized results dividing total results, with unit of %.

	Subject (NN/SVM)							
Features	A	B	C	D	E	F	G	Average
RMS	70.2/74.1	68.7/68.8	80.1/81.5	77.1/78.0	72.1/73.5	70.0/71.1	69.1/71.2	72.5/74.0
DFA	79.3/83.0	80.1/84.3	82.3/87.1	81.1/85.3	79.1/81.3	75.1/81.1	77.7/80.2	79.2/83.2
WP	93.4/95.6	92.2/93.1	93.1/94.1	91.1/93.2	91.3/94.5	91.5/94.7	90.5/93.1	90.1/92.0
MM	94.8/97.0	91.1/93.0	92.1/95.8	93.3/90.3	89.1/92.1	89.3/92.4	92.3/96.4	92.1/94.3

**Table 4:** Recognition accuracy rate for forearm pronation and supination.

	Subject (WP + SVM)						
Motion	A	B	C	D	E	F	G
P/S	91.3/97.8	98.7/94.4	96.6/97.1	94.1/97.3	93.2/95.5	96.4/92.1	98.3/90.1

**Figure 3:**
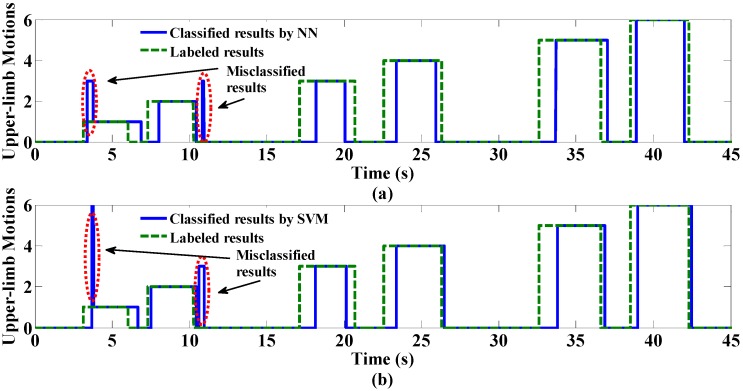
On-line testing performance with MM feature extraction method. (**a**) shows the classification results by NN; (**b**) shows the classified results by SVM. The red circles mark the misclassifications using the two classifiers.

One set of on-line testing performance data is depicted in Figure 3, where the blue lines show the classification results and the dashed green lines show the labeled results. The dotted red lines mark the misclassification results. It can be noted that the misclassification places between NN and SVM are similar.

**Figure 4:**
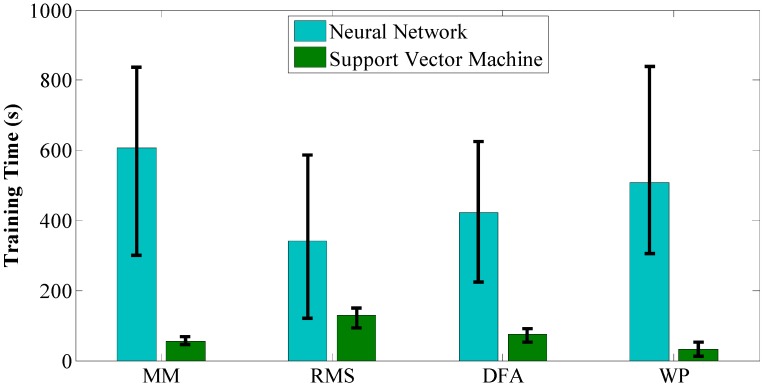
Time consumption of off-line training process. Blue bars show the average training time using NN with different features on all the subjects. Green bars show the results with SVM.

**Figure 5:**
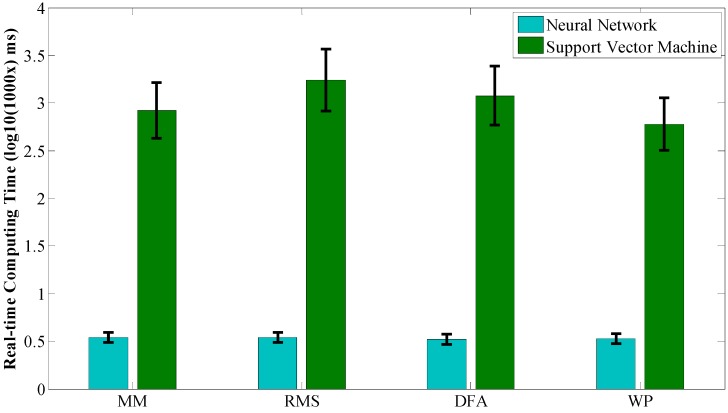
Time consumption of on-line testing process. Blue bars show the average computing time using NN with different features on all the subjects. Green bars show the results with SVM.

**Figure 6:**
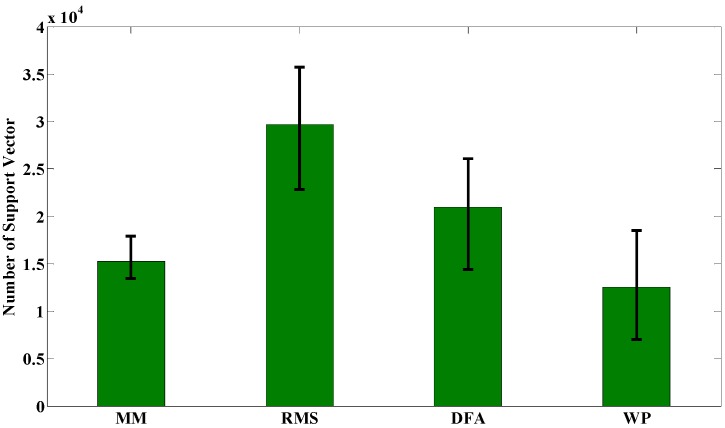
Number of support vectors for different feature extraction method.

Another important issue for motion recognition is the time consumption for training and on-line computation, while the latter is more important than the former in our particular circumstance. Experimental results for the off-line training process are depicted in Figure 4 which are the average values of off-line training times of the seven subjects on the same feature extraction method and classifier. The light blue bars denote the average training time of NN while the green bars denote the ones for SVM. It can be noticed that NN takes much more time than SVM (469.6 s *vs.* 73.5 s) to obtain a good performance. For NN, MM and WP take more time than RMS and DFA, while the former pair obtains a higher recognition accuracy rate than the latter pair. For SVM, MM and WP take less time than RMS and DFA, while the former pair still obtains a higher recognition accuracy rate than the latter pair. Experimental results for on-line calculation are depicted in Figure 5 and the average number of support vectors for different feature extraction methods are depicted in Figure 6. The blue bars denote the time consumption for one trial with NN using different feature extraction methods. There is almost no difference because the structure of the NN is the same. However, it is distinctly visible that it takes much more time for SVM to complete one recognition loop than NN (1.0918 ms *vs*. 0.0034 ms). The sampling frequency in our experiments is 1000 Hz and this is also important for sEMG sampling; a time consumption longer than 1 ms will give rise to a time delay in real-time experiments. Given this condition, RMS and DFA, with time consumptions of 1.7 ms and 1.2 ms, respectively, are not suitable for real-time purposes.

## 4. Discussion

Seven healthy subjects participated in the experiments. Although in some clinical studies a large number of participants are often used, the proposed method is going to be applied on healthy subjects and the purpose of this paper is to find the differences between these methods, not for a commercial implementation on human beings, such as in [6,7,10,11,16], so no more than nine subjects participated. Furthermore, some of these methods were widely studied for their ability of motion recognition. Given the previous consideration, a number of seven subjects was deemed sufficient for this study.

In our special circumstances of pattern recognition using sEMG signals, the experimental results of off-line training performance indicate that NN performed better than SVM on the training set of data at a cost of longer time consumption for training. However, the experimental results of real-time testing indicated that SVM is more generalized than NN on all the data combined (off-line training data and on-line testing data). As non-stationarity and time-variable properties of EMG signals, the generalization of the classifier is important for the issue of pattern recognition using EMG signals. From the experimental results of off-line training and on-line testing, it seems that NN tends to be more over-fitted to local training data while SVM tends to be relatively high in generalization abilities, which matches the respective properties of NN and SVM [9,17,25,28].

As time consumption of NN training depends on the initial values of parameters [25], the time varies from 3 min to 15 min using the MATLAB Neural Pattern Recognition Tool. In this study, the average training time for obtaining the highest recognition rate (with 1% offset) was used to represent the time consumption. Although the fastest time of NN training may be shorter than that of SVM, the performance was worse in most of the cases. During the NN training process, we found that a better training performance usually takes a longer time to train in our special cases. Also several training times are needed to obtain a relative high recognition rate because the initial parameters’ values also involve the training performance. Considering the multiple times needed for acquiring suitable training performance, the time consumption on training a NN classifier is longer than that of SVM. On the contrary, as a consequence of convex subject function, training performance of SVM is much more “stable” than NN. Using the LIBSVM, just one-time-training can obtain a good result, with shorter time consumption for training.

Nevertheless, time consumption on real-time computation of SVM is much longer than that of NN. The prediction using SVM is calculated through Equation (25) and the number of support vectors is the main factor involved in on-line time consumption. As shown in Figure 6, the number exceeds 10,000 for all four feature-extraction methods where RMS had the largest one (29,694) and WP had the smallest one (12,525). Even with the WP method, it takes 12,525 kernel function calculations to obtain one prediction result. As mentioned in Section 3.2, it is not suitable for real-time applications if the time consumption is larger than 1 ms for one prediction loop. Given this condition, a SVM, with support vector larger than 18,400, will exceed the limitations of our real-time requirement. Compared with the strategy of setting adaptive parameters, the SVM selects a large number of support vectors to guarantee the generalization on sEMG signals. Another issue for SVM in our special circumstance is that the support vector number increases with the increasing number of input training samples. As a consequence, the number of training samples cannot be too large if the SVM is applied in a real-time environment. According to our experimental results, a maximum of about 50,000 training samples will fit the requirement. This issue may be addressed by the nonlinear feature extraction method as motioned in the work of Ju *et al*. [6]. The nonlinear method has the ability to catch the dynamic information relating EMG signals and motions. Although only recognition accuracy rate was discussed in their work, it could be believed that the nonlinear method will extract the more generalized features from the data and reduce the number of support vectors.

Among the four feature extraction methods, WP obtains the highest recognition accuracy rate (97.6%) compared to 95.0% for NN and SVM, respectively, while the second one is MM with 95.1% and 92.6% for NN and SVM, respectively. RMS and DFA cannot achieve a recognition accuracy rate above 90%. One important difference between the four feature extraction methods is the adoption of a low-pass filter or low-pass-filter like algorithm. Among the feature extraction methods, MM applies a low-pass Butterworth filter directly in the processing [23]. The changing muscle force frequency is much lower than that of EMG signals due to the structure of muscle and surrounding tissue. The adoption of a low-pass filter in MM is to mimic the low-pass-filter like function of muscles. The musculotendon force gives rise to human motion while EMG signals are more like reference commands. As a consequence, it is reasonable to process features with a low-pass filter to make them more like musculotendon-force-like signals. Although no low-pass filter is used directly in WP, the algorithm of WP itself is low-pass-filter like, which tends to extract the trend of sEMG signals [25]. RMS can also be considered as a low-pass filter but the ‘cutoff’ frequency is higher than that of MM or WP and it depends on the time interval, which also involves the extraction of detail information in sEMG signals and real-time ability during application. A long time interval will eliminate useful information and cause time delays in real-time applications. As a limitation of the algorithm, DFA is hard to apply in a real-time environment.

As a consequence, for feature extraction, WP and MM performed better than the other two in our case, while MM was more effective than WP in real-time experiments. Although NN took much more training time than SVM, it took much less in real-time performance. If low time consumption is an important requirement, the combination of MM and NN is recommended. Otherwise, the combination of MM and SVM would be a good choice.

Another interesting point is that the motion of forearm pronation and supination was recognized in an indirect way, *i.e*., the features were not extracted from the muscles responding directly to the motion. *Pronator teres*, *pronator quadratus*, *supinator* and *biceps brachii* are the four muscles involved in the forearm pronation and supination motion. They are in the deep layer of the forearm, making them impossible to detect by surface electrodes. As a consequence, the motion was recognized via the synergetic behavior of muscles. Similar studies about recognizing motion using muscles around the forearm muscles can be found in [12,13,14,15,16]. In this paper, activations of the selected six muscles are quite different among individuals during the motion of forearm pronation and supination. Recognition of accuracy rates of this motion are thus quite different among individuals. However, all the recognition accuracy rates are above 90%, indicating that the applied method has the ability to perform motion recognition with the indirectly involved muscles.

## 5. Conclusions

In this paper, eight combinations of four feature extraction methods and two classifiers were tested to recognize eight human upper-limb motions, using features extracted from sEMG signals as inputs. The comparative experimental results show that features extracted by WP and MM can lead to the highest recognition accuracy rate among the four feature extraction methods, while MM is more effective in real-time applications with a trade-off of about a 3% lower recognition accuracy rate than WP. For classifiers, although NN can obtain higher recognition accuracy rate than SVM in the training process, SVM is more accurate than NN in on-line tests. However the time consumption for SVM requires the user to choose the training samples carefully and restrict the number of support vectors or it will cause time-delays in real-time applications. As a consequence, the combination of MM and NN is recommended when strict real-time performance is required. Otherwise, the combination of MM and SVM may provide higher and more stable recognition accuracy rates. The experimental results also indicate that a low-pass filter with cut-off frequency around 1–4 Hz applied in feature extraction will be helpful for increasing recognition accuracy rates.

In the future, MM will be improved to enhance the ability of resistance to the non-stationarity and time-variable properties of sEMG signals, in order to reduce the number of support vectors in SVM and improve the robustness for practical applications. These methods will be applied for robot control and rehabilitation training in the future.
